# ﻿Morphological and molecular identification of two new *Marasmiellus* species (Omphalotaceae, Agaricales) from Thailand

**DOI:** 10.3897/mycokeys.109.129791

**Published:** 2024-09-24

**Authors:** Wenhua Lu, Pumin Nutaratat, Jaturong Kumla, Saowaluck Tibpromma, Abdallah M. Elgorban, Samantha C. Karunarathna, Nakarin Suwannarach

**Affiliations:** 1 Department of Biology, Faculty of Science, Chiang Mai University, Chiang Mai 50200, Thailand; 2 Center of Excellence in Microbial Diversity and Sustainable Utilization, Department of Biology, Faculty of Science, Chiang Mai University, Chiang Mai 50200, Thailand; 3 Department of Biology, Faculty of Science and Digital Innovation, Thaksin University, Pa Phayom, Phatthalung 93210, Thailand; 4 Microbial Technology for Agriculture, Food and Environment Research Center, Faculty of Science and Digital Innovation, Thaksin University, Pa Phayom, Phatthalung 93210, Thailand; 5 Office of Research Administration, Chiang Mai University, Chiang Mai 50200, Thailand; 6 Center for Yunnan Plateau Biological Resources Protection and Utilization, College of Biological Resource and Food Engineering, Qujing Normal University, Qujing, Yunnan 655011, China; 7 Center of Excellence in Biotechnology Research (CEBR), King Saud University, Riyadh, Saudi Arabia

**Keywords:** Basidiomycota, new taxa, omphalioid mushroom, taxonomy, wood-decaying mushroom

## Abstract

*Marasmiellus* (Omphalotaceae, Agaricales) specimens collected in Thailand were investigated based on morphological characteristics and molecular phylogenetic analyses. In the present study, two species are introduced as new to science, namely *Marasmiellusthailandicus* and *M.minutisporus*. Phylogenetic analyses were carried out based on the internal transcribed spacer (nrITS) and nuclear ribosomal RNA large subunit (nrLSU) regions, and the results revealed that the two new taxa are distinct species within *Marasmiellus*. Another specimen was identified as *M.scandens* and is reported for the first time with morphology and molecular data from Thailand. Descriptions, illustrations, and phylogenetic results are provided. In addition, *M.diaphanus* and *M.colocasiae* are proposed as new combinations of *Collybiopsisdiaphana* and *Paramarasmiuscolocasiae*, respectively, based on the phylogenetic evidence.

## ﻿Introduction

The genus *Marasmiellus* Murrill was proposed by Murrill (1915), with the type species *M.juniperinus* Murrill [treated as *Collybiopsisjuniperina* (Murrill) R.H. Petersen] (Petersen and Hughes 2021). This is one of the well-known and widely distributed genera, which belongs to the family Omphalotaceae Bresinsky, order Agaricales Underw. with a worldwide distribution in the tropics and the subtropics (Moncalvo et al. 2002; Wilson and Desjardin 2005; Blanco-Dios 2015; Sesli et al. 2018). Most species of this genus are saprobes occurring on decaying plant matter, which degrade leaf and woody debris, some species are host-specific, parasitic, and attack various economically important plants (e.g., banana, coconut, and sugar cane) (Singer 1973; Jabeen et al. 2023). *Marasmiellus* species are characterized by collybioid, omphalioid, or pleurotoid basidiomata, slightly decurrent, pileus white, yellow, pink or brown, convex, lamellae well-developed, intervenose, adnate to decurrent, and smooth, white or hyaline, thin-walled, and ellipsoid to oblong, rarely subcylindrical, fusiform, inamyloid basidiospores, usually with confluent hilar appendage, and pileipellis or stipitipellis with a Rameales-structure (Singer 1973; Pérez-De-Gregorio et al. 2011). Prior to this study, eight *Marasmiellus* species, including *M.albofuscus* (Berk. & M.A. Curtis) Singer (Chandrasrikul et al. 2011), *M.alliiodorus* (Mont.) Singer (Chandrasrikul et al. 2011), *M.amygdalosporus* Pegler (Chandrasrikul et al. 2011), *M.candidus* (Fr.) Singer (Seephueak et al. 2018), *M.chamaecyparidis* (Hongo) Hongo (Chandrasrikul et al. 2011), *M.collybioides* (Speg.) J.S. Oliveira, *M.corticum* Singer (Seephueak et al. 2018), and *M.paspali* (Petch) Singer (Chandrasrikul et al. 2011) have been reported from Thailand.

Some phylogenetic studies on Omphalotaceae based on nrITS and nrLSU in single and multigene analyses have been conducted to solve the stable placement where *Gymnopus* (Pers.) Gray and *Marasmiellus* still remain uncertain. Initially, *Gymnopus* and *Marasmiellus* were treated as multiple branches, nonmonophyletic groups (Moncalvo et al. 2002; Mata et al. 2004; Wilson and Desjardin 2005; Hughes et al. 2010; Petersen and Hughes 2017). Mata et al. (2004) argued for proposing the broad concept of *Gymnopus* based on nrITS analysis which places the type species of *Marasmiellus* within a *Gymnopus* clade. However, Wilson and Desjardin (2005) argued for keeping *Marasmiellus* and *Gymnopus* as distinct genera. In addition, Oliveira et al. (2019) provided consistent evidence for more restricted *Gymnopus* and a distinct *Marasmiellus* s. str., which is composed of Gymnopussect.Vestipedes and members of Marasmiellussect.Dealbati, *Marasmiellus*, *Rameales*, and *Stenophylloides*.

According to the recent nomenclature, the type species of *Marasmiellus* (*M.juniperinus* Murrill) was transferred to *Collybiopsis* (J. Schröt.) Earle. Petersen and Hughes (2021) considered *Marasmiellus* as a synonym of *Collybiopsis*, while the concepts of *Collybiopsis* between Oliveira et al. (2019) and Petersen and Hughes (2017) are different. In addition, the boundaries among *Gymnopus*, *Marasmiellus*, and *Collybiopsis* would be more blurred, especially between *Marasmiellus* and *Collybiopsis*, as well as if these species were transferred to *Collybiopsis*, then it would be polyphyletic with *Rhodocollybia* Singer, *Paragymnopus* J.S. Oliveira, and *Lentinula* Earle would be a synonym of *Gymnopus*. Nevertheless, further clarifications are needed for whether *Collybiopsis* accommodates all members of *Marasmiellus*. In this study, we treat *Marasmiellus* following Singer’s (1974, 1986) concept.

The present study aims to describe two new *Marasmiellus* species and a new report of *M.scandens* (Massee) Dennis & D.A. Reid collected from Thailand based on morphological characteristics and multigene phylogenetic analyses. In addition, we propose two new combinations of *Collybiopsisdiaphana* and *Paramarasmiuscolocasiae*. Descriptions, illustrations, and a phylogenetic tree to show the placement of the taxa are provided.

## ﻿Materials and methods

### ﻿Morphological study

Fresh basidiomata were collected from the northern part (Chiang Mai University, Chiang Mai Province) and the southern part (Phatthalung Province) of Thailand. Macromorphological features of the basidiomata were documented and photographed in the field. Color names and codes were determined following Kornerup and Wanscher (1978). The specimens were taken back to the laboratory, dried completely in an electric oven at 45 °C (Hu et al. 2022), and sealed in plastic bags for further micro-morphological characterization. The holotype and other examined specimens were deposited at the Chiang Mai University Biology Department Herbarium (CMUB), Chiang Mai University, Thailand. Freehand sections of the dried specimens were mounted in 5% KOH and Congo red to observe microscopic characteristics, while Melzer’s reagent was used to increase the contrast of structures and the amyloid reaction of basidiospores. The light Eclipse 80i microscope (Olympus, Japan) was used to view features of basidia, basidiospores, cystidia, and hyphae, which were drawn by using the drawing tube attached to the microscope. The sizes of micro-structures were calculated based on at least 50 measurements, and the notations (a–) b–c (–d) describe the basidiospore dimensions, where the range ‘b–c’ represented 90% or more of the measured valued and a and b are the extreme values. The Q refers to the length/width ratio values of all measured basidiospores. Qm refers to the average Q value with standard deviation. ‘L’ refers to the number of complete lamellae, and ‘I’ refers to the number of lamellulae between every two complete lamellae. All the line drawings of the microstructures were made freehand based on the rehydrated materials and finally modified using Adobe Illustrator 2019.

### ﻿DNA extraction, PCR amplification, and sequencing

The genomic DNA was extracted from fresh specimens using the DNA Extraction Mini Kit (FAVORGEN, China) according to the manufacturer’s instructions. Primer pairs ITS1/ITS4 (nrITS) and LR0R/LR5 (nrLSU) regions were amplified by the polymerase chain reaction (PCR) (Vilgalys and Hester 1990; White et al. 1990), which was performed in a total volume of 20 μL reaction containing 1.0 μL DNA template, 1.0 μL each primer, 10.0 μL 2X Quick Taq® HS DyeMix (TOYOBO, Japan) and 7 μL deionized water. The amplification program was performed with an initial denaturation at 94 °C for 5 min, followed by 35 cycles of denaturation at 94 °C for 30s, annealing at 54 °C for 40 s, and an extension at 72 °C for 1 min, with a final extension at 72 °C for 10 min on a peqSTAR thermal cycler (PEQLAB Ltd., UK). PCR products were checked on 1% agarose gels stained with ethidium bromide under UV light. PCR products were purified using a PCR clean-up Gel Extraction NucleoSpin® Gel and PCR Clean-up Kit (Macherey-Nagel, Germany) following the manufacturer’s protocol. The purified PCR products were directly sequenced, and the sequences were automatically determined in the genetic analyzer at 1^ST^ Base Company (Kembangan, Malaysia) with the PCR primers mentioned above. Names of the new taxa were introduced and deposited in MycoBank (https://www.mycobank.org/, assessed on June 12, 2024). All newly generated sequences in the present study were deposited to GenBank (https://www.ncbi.nlm.nih.gov/genbank/, assessed on June 10, 2024).

### ﻿Phylogenetic analyses

The newly generated forward and reverse of sequences from this study were assembled in the BioEdit v. 7.0.5 (Hall 1999), then were subjected to BLASTn searches in the GenBank (https://blast.ncbi.nlm.nih.gov/Blast.cgi, assessed on 10 June 2024) to check those most related with a high degree of similarity taxa (with ≥ 85% query coverage and ≥ 90–100% percent identity), *Moniliophthoraperniciosa* (Stahel) Aime & Phillips-Mora (CMR UB 2041) was chosen as the outgroup from the family Marasmiaceae, and were aligned by running MAFFT v.7 at the online website server platform (www.ebi.ac.uk/Tools/mafft, assessed on June 10, 2024) (Katoh and Standley 2013) with additional sequences downloaded from GenBank and previous studies (César et al. 2020; Petersen and Hughes 2020) as is shown in Table 1. Gaps and ambiguous regions were automatically removed by trimAL v1.2 (http://trimal.cgenomics.org, assessed on June 10, 2024), and sequences were manually improved in BioEdit whenever necessary. Both sequencing datasets were combined using SequenceMatrix 1.7.8 (Vaidya et al. 2011) and AliView (Larsson 2014). The final FASTA format was converted to PHYLIP and NEXUS format in the Alignment Transformation Environment (ALTER) online program (Glez-Peña et al. 2010). The phylogenetic analyses were carried out by employing Maximum likelihood analysis (ML) and Bayesian analysis (BYPP) methods. The ML was performed on RAxML-HPC BlackBox (v.8.2.4) (Stamatakis 2014) with 1000 rapid bootstrap replicates on the CIPRES Science Gateway v.3.3 (http://www.phylo.org/portal2, assessed on 10 June 2024; Miller et al. 2010), with the GTRGAMMA substitution model. Bayesian analyses of six simultaneous Markov chains were run for 2,000,000 generations, and trees were sampled and printed to output at every 100^th^ generation (resulting in 20,000 total trees) with the GTR+I+G evolution model that was estimated using MrModeltes v. 2.2 (Nylander 2004). Phylogenetic trees were visualized using FigTree v1.4.0 (Rambaut 2009). The reliable bootstrap support values of ML (BS ≥ 60%) and BI (PP ≥ 0.90) were inserted above the nodes.

**Table 1.** Names, voucher numbers, countries, and corresponding GenBank accession numbers of the taxa used in the phylogenetic analyses of this study.

Taxa Name	Voucher	Country	GenBank Accession Number	
			nrITS	nrLSU
*Collybiopsisbiformis*	TENN58541	USA	DQ450054	NA
*C.biformis*	HMJAU61116	China	OQ597035	OQ594445
*C.brunneogracilis*	SFSU-AWW01	Indonesia	AY263434	NA
*C.carneopallida*	BRNM:747442	Italy	OM522632	NA
*C.clavicystidiata*	SFC20180705–84	South Korea	OL467252	OL462817
*C.confluens*	HMJAU61120	China	OQ597037	NA
*C.confluens*	TENN50524	Sweden	DQ450044	NA
*C.confluens*	TENN-F-067864	Germany	KP710296	NA
*C.diaphana*	Cesar202	Mexico	MT232390	NA
*C.diaphana*	Cesar44	Mexico	MT232391	NA
*C.furtive*	SFSU-F-024524h1	USA	MN413341	NA
*C.furtive*	SFSU-F-024524h2	USA	MN413342	NA
*C.istanbulensis*	KATO fungi 3596	Turkey	KX184795	KX184796
*C.juniperina*	TENN59540	USA	AY256708	KY019637
*C.juniperina*	TENN-F-58988	Argentina	KY026661	KY026661
*C.luxurians*	TENN-F-057910	USA	AY256709	AY256709
*C.luxurians*	HMJAU61101	China	OQ597045	OQ594455
*C.luxurians*	HMJAU61198	China	OQ597046	OQ594456
*C.melanopus*	SFSU AW54	Indonesia	OR818034	OR817634
*C.melanopus*	CUH AM093	India	KM896875	KP100305
*C.orientisubnuda*	NIBRFG0000500990	Turkey	OL467262	OL546546
*C.peronata*	TENN-F-065120	Belgium	KY026677	KY026677
*C.quercophila*	TENN-F-69267	Slovakia	KY026729	NA
*C.quercophila*	TENN-F-69320	USA	KY026736	NA
*C.ramealis*	TENN-F-065146	Belgium	MN413346	MW396882
*C.ramealis*	TENN-F-065145	Belgium	MN413345	MN413345
*C.ramulicola*	GDGM44256	China	KU321529	NA
*C.stenophylla*	TENN-F-051099	USA	MN413330	MW396887
*C.stenophylla*	TENN-F-065943	USA	MN413331	MW396886
*C.ugandensi*s	SFSU-BAP 614	Sao Tome	MF100986	NA
*C.vellerea*	SFC20140821-29	South Korea	OL467267	OL462810
*Gymnopusalkalivirens*	TENN51249	USA	DQ450000	NA
*G.brunneiniger*	XAL-Cesar 49	Mexico	MT232389	NG075396
*G.efibulatus*	HGASMF01-7052	China	OM970865	OM970865
*G.fusipes*	TENN59300	China	AF505777	NA
*G.fusipes*	TENN59217	France	AY256710	AY256710
*Marasmiellusagrianum*	NJ201111	Pakistan	MZ044839	NA
*M.agrianum*	NJ201112	Pakistan	MZ044840	NA
*M.alnicola*	URM90019	Brazil	KY302681	KY302682
*M.bicoloripes*	CAL1524	India	KY807129	KY817233
*M.candidus*	CBS:252.39	USA	MH856003	NA
*M.candidus*	MSM#0017	Pakistan	KJ906507	NA
*M.celebanticus*	TO HG2281	Spain	JF460781	NA
*M.gregarius*	G0197	Japan	NA	MK278330
*M.griseobrunneus*	AMH 10117	India	MK656132	MK660195
*M.griseobrunneus*	CAL 1752	India	MK660191	MK660192
*M.griseobrunneus*	AMH 10118	India	MK660194	MK660193
*M.lucidus*	s1	China	OP459424	NA
*M.lucidus*	HT10	Japan	AB968237	AB968237
***M.minutisporus***	**CMUB40054**	**Thailand**	**PP889931**	**PP890011**
***M.minutisporus***	**CMUB40055**	**Thailand**	**PP889932**	**PP890012**
*M.omphaloides*	PDD:95810	New Zealand	HQ533031	NA
*M.paspali*	AHH65	USA	EF175515	NA
*M.paspali*	AHH26	USA	EF175511	NA
*M.pilosus*	iNat91483993	Cayman Islands	OP651730	NA
*M.rhizomorphogenus*	BRNM:715003	South Korea	GU319116	GU319120
*M.scandens*	GH-80	Ghana	MN794179	NA
*M.scandens*	GH-21	Ghana	MN794139	NA
*M.scandens*	KUNCC22-12451	China	OP536418	NA
***M.scandens***	**CMUB40056**	**Thailand**	**PP889933**	**PP890013**
***M.thailandicus***	**CMUB40052**	**Thailand**	**PP889929**	**PP890009**
***M.thailandicus***	**CMUB40053**	**Thailand**	**PP889930**	**PP890010**
*M.tenerrimus*	TENN61596H1	USA	FJ596840	NA
*M.tenerrimus*	TENN61596H2	USA	FJ596841	NA
*M.tricolor*	M01452	Estonia	LR872638	NA
*M.venosus*	TNS-F-52281	Japan	AB968236	NA
*M.violaceogriseus*	PDD:95788	New Zealand	HQ533014	NA
*M.volvatus*	URM 84466	Brazil	KC348449	KC348442
*M.sacchari*	CBS:215.32	USA	NA	MH866745
*Moniliophthoraperniciosa*	CMR UB 2041	Brazil	AY317136	NA
*Paragymnopusfoliiphilus*	TENN-F-68183	USA	KY026705	KY026705
*P.perforans*	TENN-F-50318	Sweden	KY026623	KY026623
*P.perforans*	TENN-F-50319	Sweden	KY026624	KY026624
*P.pinophilus*	TENN-F-69207	USA	KY026725	KY026725
*Paramarasmiuscolocasiae*	SP376044	Brazil	GQ452780	NA
*Pa.mesosporus*	TNS-T-48339	Japan	OM522625	OM522623
*Pa.palmivorus*	AKD 112/2015	India	MG251431	MG251441
*Paramycetinisaustrobrevipes*	TENN-F-50135	Australia	KY026622	KY026622
*Par.caulocystidiatus*	TENN-F-5405	New Zealand	KY026645	NA
*Pseudomarasmiusefibulatus*	TENN-F-56187	New Zealand	MK268234	NA
*Ps.glabrocystidiatus*	BRNM 718676	Korea	NR152899	KF251093
*Ps.nidus-avis*	Cesar36	Mexico	MH560576	NA
*Ps.pallidocephalus*	TENN-F-52401	USA	KY026635	KY026635
*Ps.patagonianus*	TENN-F-54424	Chile	KY352649	NA
*Ps.quercophylloides*	TENN-F-49177	China	MK268235	NA
*Pusillomycesmanuripioides*	JO674	Brazil	MK434210	MK434211
*Pu.manuripioides*	JO1121	Brazil	MK434212	MK434213

Note: “NA” = Not available in GenBank database. Specimens obtained from this study are in bold.

## ﻿Results

### ﻿Phylogenetic analyses

**Figure 1.**
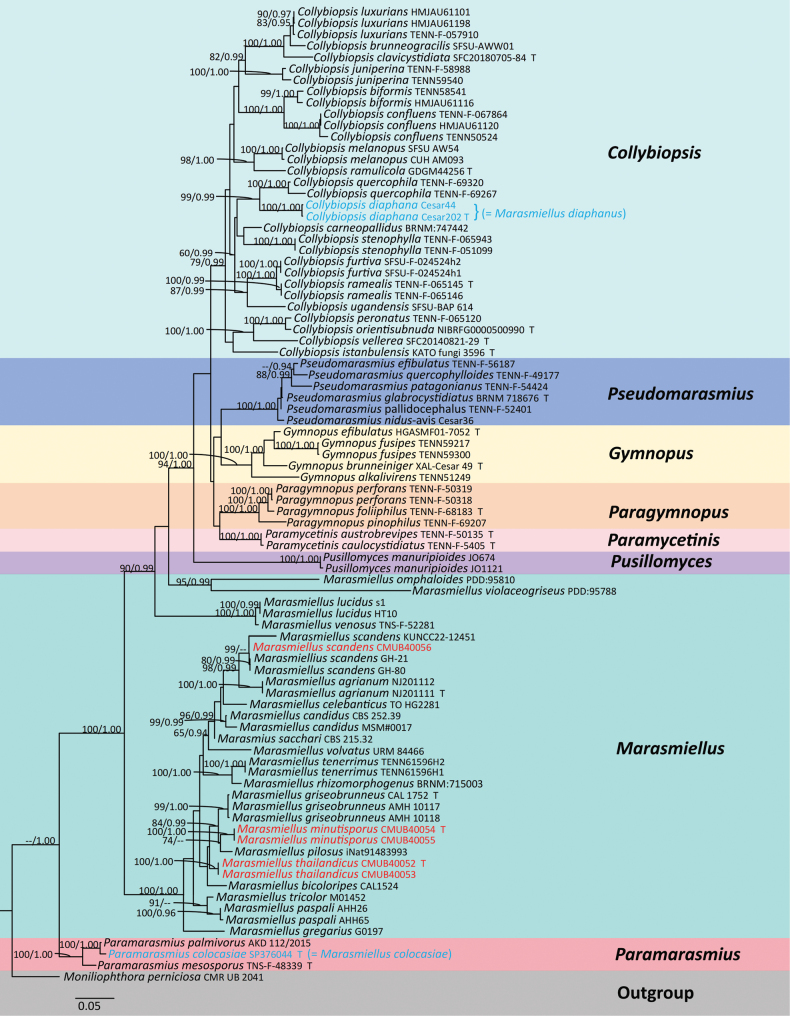
A combined phylogenetic tree was generated from maximum likelihood analysis (RAxML) based on a combined nrITS and nrLSU dataset. Bootstrap values (BS) ≥ 60% from ML analysis (left) and Bayesian posterior probabilities (right) (PP) ≥ 0.90 are shown on the branches. Newly sequenced collections are indicated in red, new combinations are in blue, and the type specimens are denoted by ‘T’.

In the dataset, the combined nrITS and nrLSU sequence dataset consisted of a total number of 86 taxa, and the aligned dataset was comprised of 1593 characters, including gaps (nrITS: 1–709 and nrLSU: 710–1593). The best RAxML tree was obtained with a final ML optimization likelihood value of -15536.515830. The matrix had 780 distinct alignment patterns, with 34.45% undetermined characters or gaps. Estimated base frequencies were as follows: A = 0.248165, C = 0.187111, G = 0.251637, T = 0.313087, with substitution rates AC = 1.027131, AG = 5.242142, AT = 1.916708, CG = 0.743477, CT = 6.311829, GT = 1.000000; gamma distribution shape parameter *α* = 0.681313, tree-Length = 3.382466. Notably, the phylograms of the ML and BI analyses were similar in topology. Therefore, the phylogenetic tree obtained from ML analysis was selected and is presented in this study (Fig. 1).

Two specimens in this study, CMUB40054, and CMUB40055, were clustered in a well-supported lineage (1.00 PP/100% BS), introduced as *M.minutisporus* related to *M.pilosus* (Dennis) Singer (iNat91483993), and sister to *M.griseobrunneus* Sharafudheen & Manim (AMH 10117, AMH 10118, and CAL1752) with a support value of 0.99 PP and 84% BS). The second species of this study (CMUB40052 and CMUB40053) formed a separate lineage within the genus *Marasmiellus* with higher support values (1.00 PP/100% BS) and formed a clade with *M.bicoloripes* K.P.D. Latha, K.N.A. Raj & Manim (CAL1524) and *M.minutisporus* cluster, and we introduced it as *M.thailandicus*. Additionally, one collection (CMUB40056) was clustered with *M.scandens* strain (GH80 and GH21) with strong statistical support (0.99 PP/100% BS). Noteworthily, *M.scandens* (KUNCC22-12451) differs from *M.scandens* (GH21) and (GH80) by 29 base pair differences out of 577, but our strain (CMUB40056) only has two base pair differences out of 570 bases (Fig. 1). Additionally, the phylogenetic tree showed that *M.diaphanus* César, Bandala & Montoya (Cesar 202 Type, and Cesar 44) belonged to *Collybiopsis* and formed a sister taxon to *C.quercophila* (Pouzar) R.H. Petersen (TENN-F-69320 and TENN-F-69267) with high statistical support (1.00 PP/99% BS; Fig. 1). *Marasmielluscolocasiae* Capelari & Antonín (SP376044, Type) assigned to the *Paramarasmius* and formed a sister taxon to *P.palmivorus* (Sharples) Antonín & Kolařík (AKD 112/2015) and clustered with *P.mesosporus* (Singer) Antonín, K. Hosaka & Kolařík (TNS-F-48339, Type) with strong support value (1.00 PP/98% ML; Fig. 1) in *Paramarasmius* Antonín & Kolařík branch. Therefore, based on phylogenetic analyses, *M.diaphanus* is proposed as *Collybiopsisdiaphana* comb. nov., while *M.colocasiae* is proposed as *Paramarasmiuscolocasiae* comb. nov.

### ﻿Taxonomy

#### ﻿ Marasmiellus thailandicus

Taxon classificationFungiAgaricalesOmphalotaceae

W. Lu, N. Suwannar. & J. Kumla sp. nov.

7B1E4EBC-59D7-5778-BF25-0C5434A843ED

854272

Figs 2A, B , 3

##### Type.

Thailand • Chiang Mai Province, Chiang Mai University; 18°48'5"N, 98°57'23"E; elevation 337 m; on bark of *Lagerstroemiamacrocarpa* Wall. ex Kurz, 5 August 2023, N. Suwannarach & J. Kumla (CMUB40052). GenBank accession numbers PP889929 (nrITS) and PP890009 (nrLSU).

##### Etymology.

“*thailandicus*” refers to the country Thailand, where the type species was collected.

##### Diagnosis.

Differs from *M.candidus* by the presence of a reddish gray to dull red, dry, radially wrinkled surface, grooved pileus with distant lamellae, and obovate or ellipsoid spores.

##### Macrostructures.

***Basidiomata*** small-sized, marasmioid. ***Pileus*** 6–20 mm diam., hemispherical first, expanding to plano-convex with slightly concave, radially sulcate with age, with involute then deflexed or straight margin, not distinctly hygrophanous, translucently striate up to center, reddish gray (10B2) to dull red (10B3) all over with grayish red (7B3) center, grayish red (7B3) margin, surface smooth, dry, and dull. ***Lamellae*** distant, broadly adnate to subdecurrent, sometimes anastomosing, white to cream-colored, with a reddish white (7A2) edge, particularly in young specimens, I = 2–3, L = 11–14. ***Stipe*** 4–8 × 1–2 mm, cylindrical, curved, base often slightly swollen, inconspicuous fibrils or scurfy, creamy-white at apex, slightly reddish white (7A2) at the base, dry, pruinose all over, ***Context*** thin, fistulose or solid, concolorous with surface. ***Smell and taste*** indistinct.

**Figure 2.**
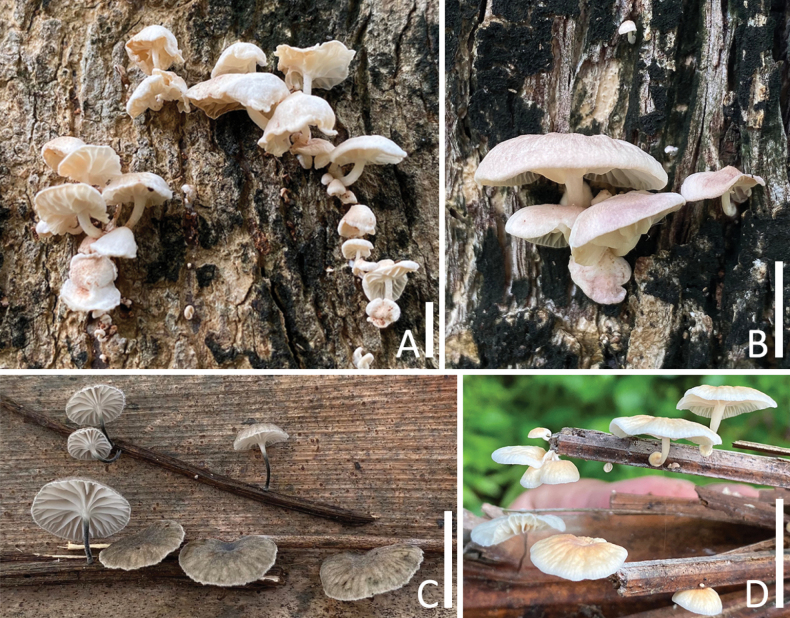
Habitat of *Marasmiellus* species in the present study **A**, **B***Marasmiellusthailandicus* (CMUB40052, holotype) **C***Marasmiellusminutisporus* (CMUB40054, holotype) **D***Marasmiellusscandens* (CMUB40056, new record). Scale bars: 10 mm (**A–D**).

##### Microstructures.

***Basidiospores*** (–12)13–16(–17) × 4–5(–6) μm (average = 15 × 5 μm), Q = (2.1)2.5–3.5(–3.75), Qm = 3.05 ± 0.38, sub-cylindrical to elongate with apiculus, inamyloid, thin-walled. ***Basidia*** 20–45 × 7–11 μm, 4-spored, clavate, sterigmata up to 5 μm. ***Cheilocystidia*** 50–53 × 8–13 μm, clavate. ***Pleurocystidia*** absent. ***Trama hyphae*** cylindrical, thin-walled, hyaline, inamyloid. ***Pileipellis***, a cutis with transitions to a trichoderm, made up of cylindrical; pigment brown, intracellular and minutely incrusting, hyphal 2–4 μm, negative in Melzer’s reagent. ***Stipitipellis***, a cutis of parallel, somewhat skewed, cylindrical, or clavate-shaped, smooth hyphae, thin-walled, up to 7.5 um wide. ***Caulocystidia*** absent. ***Clamp connection*** present.

**Figure 3.**
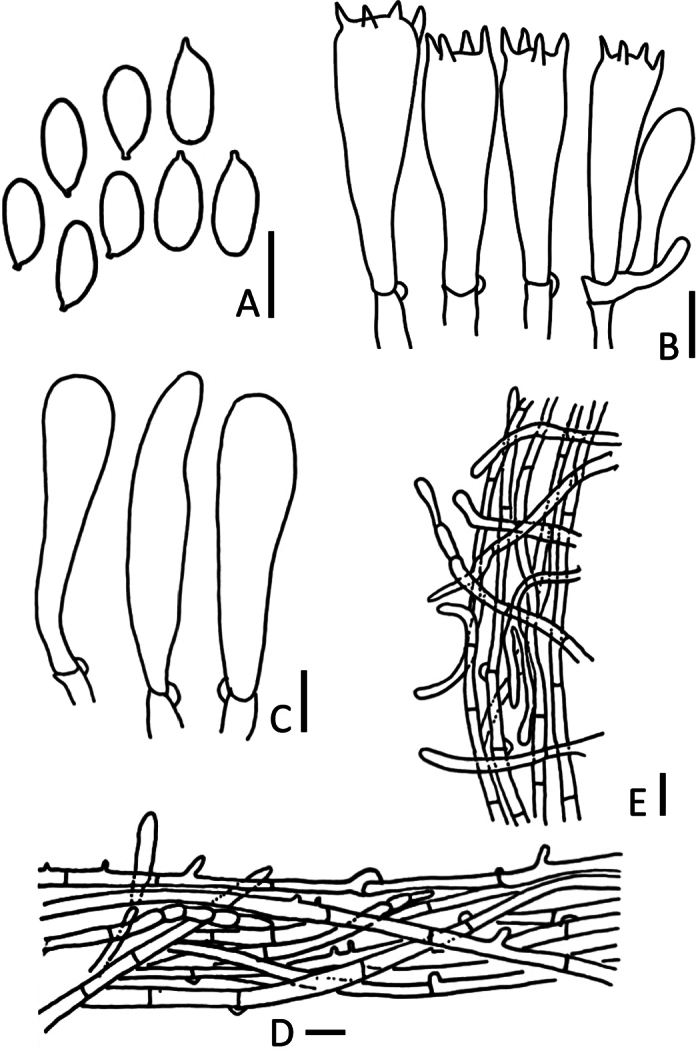
*Marasmiellusthailandicus* (CMUB40052, **holotype**) **A** basidiospores **B** basidia **C** cheilocystidia **D** terminal elements of pileipellis **E** hyphae of stipe. Scale bars: 10 μm (**A–E**).

##### Ecology and distribution.

Caespitose, in small groups growing on the bark of *Lagerstroemiamacrocarpa*. Known only from the type locality in northern Thailand.

##### Additional material examined.

Thailand, • Chiang Mai Province, Chiang Mai University; 18°48'5"N, 98°57'23"E; elevation 337 m; on bark of *Lagerstroemiamacrocarpa*, 24 August 2023, N. Suwannarach & J. Kumla (CMUB40053). GenBank accession numbers PP889930 (nrITS) and PP889930 (nrLSU).

#### ﻿ Marasmiellus minutisporus

Taxon classificationFungiAgaricalesOmphalotaceae

W. Lu, P. Nutaratat & J. Kumla sp. nov.

A118B6C4-CD3B-5220-A2EC-0E1EED789A62

854273

Figs 2C , 4

##### Type.

Thailand • Phatthalung Province, Khuan Khanun, Sago Palm (*Metroxylonsagu*) Forest, 7°44'02"N, 99°59'47"E; elevation 23 m; on decaying leaf and branches of deciduous tree; 6 September 2023; P. Nutaratat, P., Suwannarach & J. Kumla, (CMUB40054). GenBank accession numbers PP889930 (nrITS) and PP890011 (nrLSU).

##### Etymology.

“*minutisporus*” refers to the small basidiospores of this species.

##### Diagnosis.

Differs from *M.virgatocutis* by the grayish-brown, convex, wrinkled pileus, longer pileus terminal, smaller elongated spores, and caulocystidia.

##### Macrostructures.

***Basidiomata*** small-sized, marasmioid. ***Pileus*** 5–11 mm diam., thin, then expanding to applanate, with slightly inflexed, pulvinate when young, then deflexed, finally reflexed, and undulating margin, convex when age with depressed to umbilicate at disc., grayish brown (7D3), often with grey, brown, or dark gray (1F1) at the center, gray at the margin; often radially wrinkled, surface dry, slightly pruinose to tomentose under the lens. ***Lamellae*** distant, often more or less reduced, white to sordid beige with a concolorous overall, pruinose edge, I = 1–3, L = 13–16. ***Stipe*** 4–9 × 1 mm, cylindrical, often subbulbous at the base, off-white at the apex, fourth to fifth downward, and black or gray at the stipe base, entirely white pruinose, with basal tomentum. ***Context*** thin, soft, white, fistulose. ***Smell and taste*** none.

**Figure 4.**
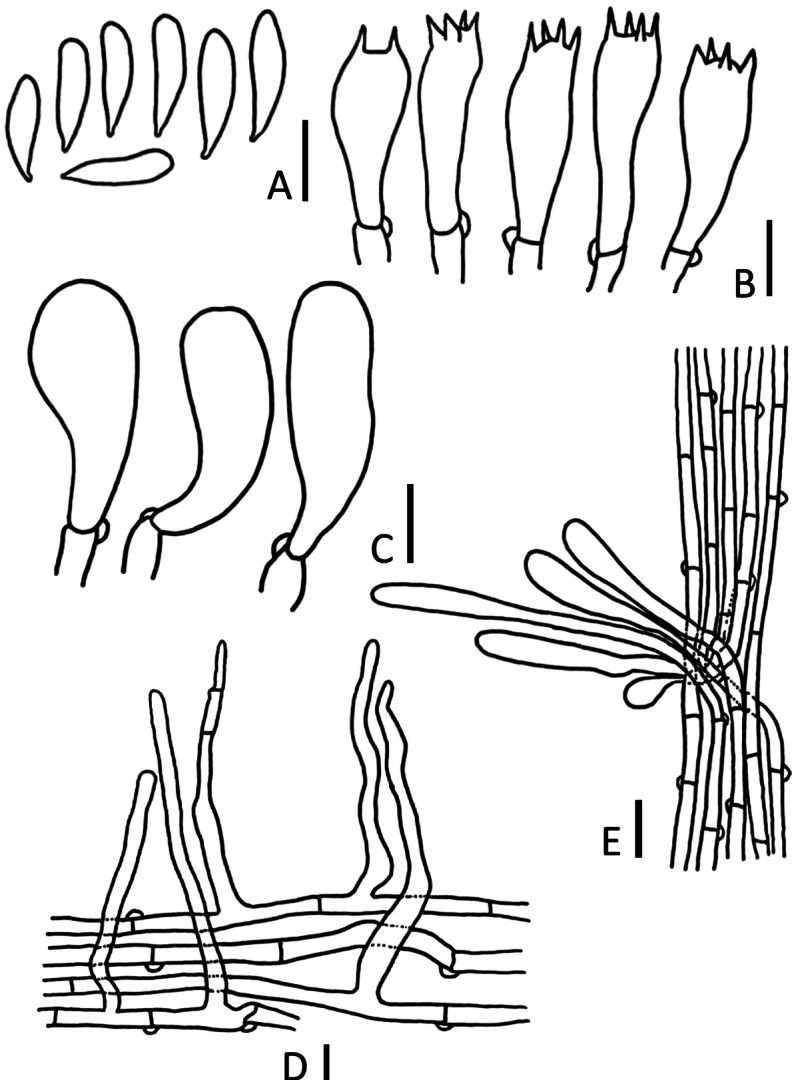
*Marasmiellusminutisporus* (CMUB40054, **holotype**) **A** basidiospores **B** basidia **C** cheilocystidia **D** elements of pileipellis **E** hyphae elements of stipe and caulocystidia. Scale bars: 5 μm (**A**); 10 μm (**B–E**).

##### Microstructures.

***Basidiospores*** (11)12–15(–16) × (3)4–5 μm (average = 14 × 4 μm), Q = (2.6)2.8–4(4.67), Qm = 3.5 ± 0.56, hyaline, inamyloid, cylindrical to fusiform, thin-walled. ***Basidia*** 25–28 × 7–9 μm, 4-spored, sterigmata up to 5 μm long, clavate. ***Lamella edge*** sterile. ***Cheilocystidia*** 30–33 × 12–15 μm, somewhat clavate to subglobose, ***Pleurocystidia*** absent. ***Pileipellis*** a cutis made up of 4.0–10 μm wide, inamyloid, inflated or cylindrical hyphae, with scattered suberect to erect, thin-walled, smooth, sometimes terminal elements up to 61–99 × 3.0–10 μm, gradually tapering to an acute or rounded apex. ***Stipitipellis*** a cutis composed of cylindrical, inamyloid, parallel, slightly, not incrusted, smooth, thick-walled. ***Caulocystidia*** 25–50 × 4–5 μm, adpressed to erect, cylindrical, clavate, smooth, thin-walled. ***Clamp connection*** present.

##### Ecology and distribution.

Solitary to caespitose, in small groups growing on decaying leaves and twigs of deciduous trees. Known only from the type locality in southern Thailand.

##### Additional material examined.

Thailand • Phatthalung Province, Khuan Khanun, Sago Palm (*Metroxylonsagu*) Forest, 7°44'02"N, 99°59'46"E; elevation 23 m; on decaying leaf and twigs of deciduous tree; 7 September 2023; P. Nutaratat, N. Suwannarach & J. Kumla, (CMUB40055). GenBank accession numbers PP889932 (nrITS) and PP890012 (nrLSU).

#### ﻿ Marasmiellus scandens

Taxon classificationFungiAgaricalesOmphalotaceae

(Massee) Dennis & D.A. Reid, Kew Bull. [11](2): 289 (1957)

B9AF3E69-5E94-518E-94A8-B3649C33B8EB

300136

Figs 2D , 5

##### Macrostructures.

***Basidiomata*** small-sized, marasmioid. ***Pileus*** 5–10 mm diam., orbicular when young and then convex at age, streaked from disc to margin, margin entire, wavy to irregular, decurved or greatly reflexed, surface dry, smooth, white pruinose, white to grayish orange (5B4–5), grayish orange (5B4) at the margin. ***Lamellae*** adnate, subdistant, with 2–3 series of lamellulae, 14–16 major lamellae, unequal, narrow, pale white at face and edge. Smell and taste none. ***Stipe*** 4–6 × 1–2 mm, often curved, lateral or central, disc at the base, dry, surface smooth, whitish to pale orange (5A3); Context thick, fistulose, orange white (5A2–3).

**Figure 5.**
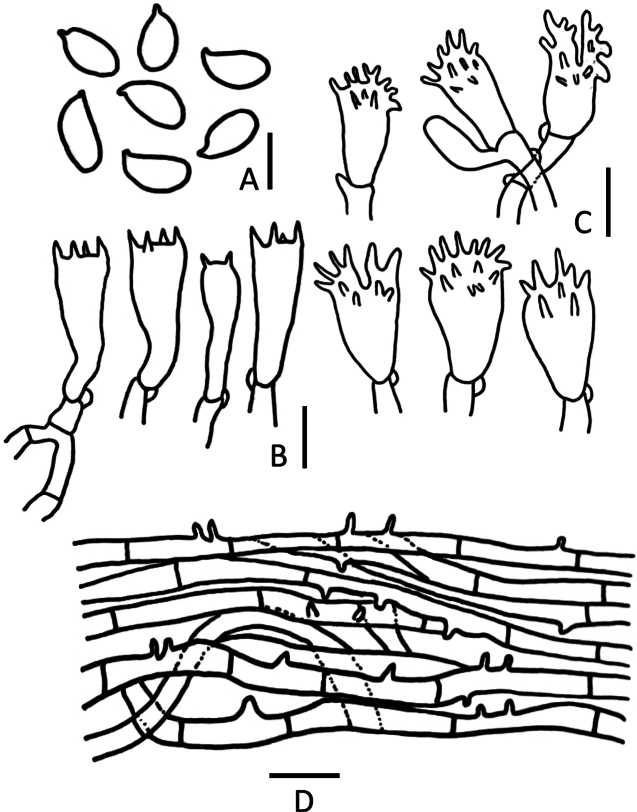
*Marasmiellusscandens* (CMUB40056, new record) **A** basidiospores **B** basidia **C** cheilocystidia **D** terminal elements of pileipellis. Scale bars: 5 µm (**A–C**); 10 µm (**D**).

##### Microstructures.

***Basidiospores*** 7–9(–10) × (3)3.5–4.5(–6) µm (average = 8 × 4.5 μm), Q = 1.4–1.8(–2), Qm = 1.74 ± 0.11, broadly ellipsoid, smooth, hyaline, inamyloid, thin-walled. Basidia 10–12.5 × 3–4.5 µm, clavate, 4-spored, sterigmata up to 1.5 µm. ***Cheilocystidia*** abundant, 15–17(–22) × 8–9(–11) µm, broom-cell type with finger-like excrescences in the upper half, hyaline, thin-walled. ***Pleurocystidia*** absent. ***Pileipellis*** with poorly ***Ramealis***-structure, ***trama*** hyphae 4–9 µm, negative in Melzer’s reagent. ***Stipitipellis*** hyphae up to 5 μm wide, smooth, thin-walled, ***trama*** not observed, negative in Melzer’s reagent. ***Clamp connections*** present.

##### Ecology and distribution.

Caespitose, in small groups on decaying leaf of *Metroxylonsagu* in southern Thailand (This study), on *Aquilariasinensis* (agarwood) trees in China (Zhang et al. 2023), and on the stem of mangosteen in Malaysia (Turner 1971), on cocoa in Australia, Ghana, Caribbean, Papua New Guinea, and Solomon Islands (Amoako-Attah et al. 2020; Ali et al. 2021), on coffee in Fiji, Guinea, and Sierra Leone (Lu et al. 2022).

##### Material examined.

Thailand • Phatthalung Province, Khuan Khanun, Sago Palm (*Metroxylonsagu*) Forest, 7°44'02"N, 99°59'47"E; elevation 24 m; on decaying leaf of *Metroxylonsagu*; 3 September 2023; P. Nutaratat, N. Suwannarach & J. Kumla, (CMUB40056). GenBank accession numbers PP889933 (nrITS) and PP890013 (nrLSU).

### ﻿New combinations

#### ﻿ Collybiopsis diaphana

Taxon classificationFungiAgaricalesOmphalotaceae

(César, Bandala & Montoya) W. Lu, N. Suwannar. & J. Kumla comb. nov.

2DE07919-8D25-56CE-AB7D-4BC1778BD9A8

854271

≡ Marasmiellusdiaphanus César, Bandala & Montoya, in César, Montoya, Bandala & Ramos, *Mycol. Progr.* 19(10): 1022 (2020)

#### ﻿ Paramarasmius colocasiae

Taxon classificationFungiAgaricalesOmphalotaceae

(Capelari & Antonín) W. Lu, N. Suwannar. & J. Kumla comb. nov.

06300D81-DFF1-5B72-A400-159BAA4FC461

854270

≡ Marasmielluscolocasiae Capelari & Antonín, *Cryptog. Mycol.* 31(2): 138 (2010)

## ﻿Discussion

A combination of morphological characteristics and molecular phylogeny inferred from sequence data of the nrITS and nrLSU region revealed two new species of *Marasmiellus* (*M.thailandicus* and *M.minutisporus*) and one known species (*M.scandens*) in this study. According to the phylogeny results, *Marasmiellus* is well-clustered in a strongly supported clade (1.00 PP/100% BS) (Fig. 1), which is consistent with the results of previous phylogenetic studies (Sesli et al. 2018; Sharafudheen and Manimohan 2019; César et al. 2020). Furthermore, the phylogenetic tree indicated that *M.diaphanus* and *M.colocasiae* belonged to *Collybiopsis* and *Paramarasmius*, respectively. Therefore, these two new combinations were introduced as *C.diaphana* and *P.colocasiae*.

Morphologically, *M.thailandicus* is closely related to *M.subnigricans* (Murrill) Singer, *M.candidus*, and *M.bicoloripes*. *Marasmiellussubnigricans* (11−45 mm diam.) and *M.candidus* (3–22 mm diam) contain dingy cream and purely white to sordid white (Antonín and Noordeloos 2010; Retnowati 2018). However, *M.subnigricans* differs from *M.thailandicus* by having smaller basidia (24−32 × 8−8.8 μm), hygrophanous, subtranslucent to strongly translucent pileus, and longer stipe (13−50 × 1−3 mm), and larger basidiospores (13.6−17.6 × 4−5.6 μm). *Marasmielluscandidus* has white grooved, large pileus with a tendency to develop pinkish tinges at age, brown and swollen at the base, long, slender neck cheilocystidia, and caulocystidia, as well as large pileus (Antonín and Noordeloos 2010). *Marasmiellusbicoloripes* is distinguished from *M.thailandicus* by having smaller basidiospores (4−8 × 3−4 μm) and pileipellis without setae. Phylogenetically, *M.thailandicus* formed a monophyletic clade and separated other *Marasmiellus* species with strong statistical support.

Based on morphological characteristics, *M.minutisporus* is closely related to *M.virgatocutis* Robich, Esteve-Rav. & G. Moreno and *M.griseobrunneus*. However, *M.virgatocutis* is distinguished by the radially fibrillose-virgate pileus surface and the variable shape of cheilocystidia, which range from clavate to lageniform to molariform. While *M.griseobrunneus* has a surface of dark brown, the largest pileus (8−38 mm diam.), and observed pleurocystidia compared with *M.minutisporus* (Sharafudheen and Manimohan 2019). Phylogenetically, *M.minutisporus* is closely related to *M.pilosus*. However, *M.minutisporus* differs from *M.pilosus* in that it is grayish-brown, convex, wrinkled pileus with a fimbriate margin and the absence of pleurocystidia (Singer 1973).

*Marasmiellusscandens* was previously reported worldwide (in Asia, Africa, Oceania, and North America) as a pathogen and endophytic form in cocoa and coffee (Amoako-Attah et al. 2020; Ali et al. 2021; Lu et al. 2022; Zhang et al. 2023). In this study, the Thai specimen is similar to the descriptions of Dennis and Reid (1957) from *M.scandens*, originally described as *Marasmiusscandens* by Massee (1910). However, Thai specimens mostly differed from Chinese specimens (HKAS-124582) (Zhang et al. 2023) in the shape of cheilocystidia with a broom-cell type. This may be influenced by the phenotypic variability across a wide geographic range.

Prior to the present study, eight species of *Marasmiellus* have been reported based on morphological characteristics in Thailand viz. *M.albofuscus*, *M.alliiodorus*, *M.amygdalosporus*, *M.candidus*, *M.chamaecyparidis*, *M.collybioides*, *M.corticum*, and *M.paspali* (Chandrasrikul et al. 2011; Seephueak et al. 2018). Most of them are widely found in Asia (China, Indonesia, and Japan), North America (Hawaii and Florida), South America (Argentina, Brazil, and Chile), the Caribbean (Cuba and the Lesser Antilles), and Oceania (Hongo 1975; Jackson and Firman 1982; Kohler et al. 1997). Some are only known in South America (*M.alliiodorus*) and West Africa (*M.paspali*) (Spegazzini 1889; Singer 1962; Pegler 1977; Buadu et al. 2002; Deng et al. 2011; Zhu et al. 2024). Historically, Thai macrofungi have been classified and described based on morphological characteristics. Of them, some are associated with the species previously known only in America, Europe, and other continents (Suwannarach et al. 2022a, b). Therefore, these eight species require further confirmation through newly collected specimens identified based on morphology and phylogeny. Additionally, this finding has increased the number of *Marasmiellus* species found in Thailand to 11. Finally, this finding is important in stimulating future studies of *Marasmiellus* in Thailand and contributes to distribution, diversity, phylogeny, and classification in Asia and worldwide.

## Supplementary Material

XML Treatment for Marasmiellus thailandicus

XML Treatment for Marasmiellus minutisporus

XML Treatment for Marasmiellus scandens

XML Treatment for Collybiopsis diaphana

XML Treatment for Paramarasmius colocasiae

## ﻿Additional information

### Author ORCIDs

Wenhua Lu https://orcid.org/0000-0001-7283-7596

Pumin Nutaratat https://orcid.org/0000-0001-7924-4822

Jaturong Kumla https://orcid.org/0000-0002-3673-6541

Saowaluck Tibpromma https://orcid.org/0000-0002-4706-6547

Abdallah M. Elgorban https://orcid.org/0000-0003-3664-7853

Samantha C. Karunarathna https://orcid.org/0000-0001-7080-0781

Nakarin Suwannarach https://orcid.org/0000-0002-2653-1913

### Data availability

All of the data that support the findings of this study are available in the main text
